# Supplementary material for: OpenMM 7: Rapid development of high performance algorithms for molecular dynamics
Source: PLoS Comput Biol. 2017 Jul 26;13(7):e1005659. doi: 10.1371/journal.pcbi.1005659 (PMC5549999; doi:10.1371/journal.pcbi.1005659)
Supplement: S1 Source Code — (ZIP) [file pcbi.1005659.s002.zip › OpenMM-7.0.1-Source/docs/api-python/generated/simtk.openmm.app.dcdfile.DCDFile.html]

DCDFile — OpenMM 7.0.1.dev-5e86c4f documentation


# DCDFile¶

*class* `simtk.openmm.app.dcdfile.``DCDFile`(*file*, *topology*, *dt*, *firstStep=0*, *interval=1*)¶
:   DCDFile provides methods for creating DCD files.

    DCD is a file format for storing simulation trajectories. It is supported by many programs, such
    as CHARMM, NAMD, and X-PLOR. Note, however, that different programs produce subtly different
    versions of the format. This class generates the CHARMM version. Also note that there is no
    standard byte ordering (big-endian or little-endian) for this format. This class always generates
    files with little-endian ordering.

    To use this class, create a DCDFile object, then call writeModel() once for each model in the file.

    `__init__`(*file*, *topology*, *dt*, *firstStep=0*, *interval=1*)¶
    :   Create a DCD file and write out the header.

        |  |  |
        | --- | --- |
        | Parameters: | - **file** (*file*) – A file to write to - **topology** (*Topology*) – The Topology defining the molecular system being written - **dt** (*time*) – The time step used in the trajectory - **firstStep** (*int=0*) – The index of the first step in the trajectory - **interval** (*int=1*) – The frequency (measured in time steps) at which states are written   to the trajectory |

    Methods

    |  |  |
    | --- | --- |
    | `__init__`(file, topology, dt[, firstStep, ...]) | Create a DCD file and write out the header. |
    | `writeModel`(positions[, unitCellDimensions, ...]) | Write out a model to the DCD file. |

    `__delattr__`¶
    :   x.\_\_delattr\_\_(‘name’) <==> del x.name

    `__format__`()¶
    :   default object formatter

    `__getattribute__`¶
    :   x.\_\_getattribute\_\_(‘name’) <==> x.name

    `__hash__`¶

    `__reduce__`()¶
    :   helper for pickle

    `__reduce_ex__`()¶
    :   helper for pickle

    `__repr__`¶

    `__setattr__`¶
    :   x.\_\_setattr\_\_(‘name’, value) <==> x.name = value

    `__sizeof__`() → int¶
    :   size of object in memory, in bytes

    `__str__`¶

    `writeModel`(*positions*, *unitCellDimensions=None*, *periodicBoxVectors=None*)¶
    :   Write out a model to the DCD file.

        The periodic box can be specified either by the unit cell dimensions
        (for a rectangular box), or the full set of box vectors (for an
        arbitrary triclinic box). If neither is specified, the box vectors
        specified in the Topology will be used. Regardless of the value
        specified, no dimensions will be written if the Topology does not
        represent a periodic system.

        |  |  |
        | --- | --- |
        | Parameters: | - **positions** (*list*) – The list of atomic positions to write - **unitCellDimensions** (*Vec3=None*) – The dimensions of the crystallographic unit cell. - **periodicBoxVectors** (*tuple of Vec3=None*) – The vectors defining the periodic box. |

High performance molecular simulation on GPUs


### Navigation

- Application Layer
  - Loaders and Setup
  - Representation and Manipulation
  - Simulation
  - Reporting Output
  - Extras
- Library Layer

©2015, Stanford University and the Authors.
|
Powered by Sphinx 1.3.1
& Alabaster 0.7.7
|
Page source
